# Recycling of platinum group metals from energy storage devices: a techno-economical business plan analysis

**DOI:** 10.12688/openreseurope.14866.1

**Published:** 2022-07-27

**Authors:** Stylianos Spathariotis, Konstantinos Miltiadis Sakkas, Ekaterini Polyzou, Iakovos Yakoumis

**Affiliations:** 1 MNLT Innovations PC, Kifisias Ave. 125-127, Athens, 11524, Greece; 2 MONOLITHOS Catalysts & Recycling Ltd., Vrilissou Str. 83., Polygono-Athens, 11476, Greece

**Keywords:** Business planning, Economics, Sustainability, Platinum Group Metals, Recycling, Innovation, Economic viability

## Abstract

**Background: **Energy storage devices utilise platinum group metals (PGMs) for their operation and exhibit an increasing adoption rate as green energy production means. Europe consumes the largest amount of PGMs worldwide (ca. 20% of the global demand), with the EU demand reaching ~71.5 t Pd, 78.7 t Pt in 2019; ~90% and 54% respectively for the production of (electro)catalysts. PGMs prices are on the rise, while these materials exhibit a risk of supply as their production in the EU is insignificant; South Africa and Russia produce approximately 84% of the global supply.

**Methods**: PGMs recycling and reutilisation from End-of-Life products to new ones, can drastically facilitate the reduction of the supply/demand gap. Innovation business planning is needed to commercialise the recovered PGMs, which includes marketing and financial elements utilised from an innovation perspective.

**Results**: SWOT (Strengths-Weaknesses-Opportunities-Threats) analysis is formed alongside a Business Model Canvas to define the Business Model and commercial added value.

**Conclusion**: The Financial Plan illustrates that a PGMs recycling facility can be profitable upon the generated market volume and assists in determining the financial sustainability by calculating the Net Present Value (NPV), Initial Rate of Return (IRR) and the break-even point for an investment made.

## Plain language summary

Primary energy production, industrial operations and certain end-applications are associated with high carbon emissions, while devices called fuel cells and electrolysers have been developed to reduce the emissions of harmful gases such as CO _2_, CO, etc. Precious metals (PGMs) are used as electrocatalysts on membrane electrode assemblies (MEAs) in order to enhance and accelerate energy production reactions. The green energy production sector grows rapidly, with industries producing green energy for their operations, while minimizing their environmental impact. Thus, a high demand is generated for PGMs, while their supply is still limited, leading to economic imbalances, specifically in the EU area. Recycling these MEA materials once the corresponding devices reach their end-of-life to obtain the contained PGMs, and the re-manufacturing/re-generation of electrocatalysts for new MEAs and fuel cells/electrolysers are major routes to ensure resource sustainability. In this study, it is shown that the aforementioned markets are expected to increase rapidly in the near future, potentially generating vast amounts of EoL MEAs. The traits of the market and the current resources are analysed to form the SWOT analysis, while the elements and characteristics of the business model of recycling the EoL MEAs and re-manufacturing electrocatalysts are presented to define a holistic model under which such operation can be profitable. Finally, a cumulative table illustrating the costs and revenues from operating business in this sector is generated. The table shows the recycling capacity for EoL MEAs and the remanufactured amount of electrocatalysts based on the recycled PGMs, the associated revenues and costs, to conclude that the profitable business margin from such operation for 5 years is estimated at EUR 4.4 million. This shows that, the rise of the green energy production market will allow for profitable business to be operated under the defined parameters, also aligned with low environmental impacts.

## Introduction

The demand for energy has rapidly increased and at the same time, due to gradual fossil fuel resources depletion, as well as climate changes issues (air pollution, global warming), there is an urgent need for renewable and environmentally friendly alternative fuels. In order to meet the growing energy needs of society Fuel Cell and Hydrogen (FCH) technologies are required, towards reducing the greenhouse gas (GHG) emissions (currently controlled by the EU Emissions Trading System (ETS), the Effort Sharing legislation and the legislation on emissions and removals from LULUCF) ^1^. Fuel cells convert chemical energy from fuels directly to electricity and heat by using electrochemical processes. They consist mainly of solid parts and their operation is noiseless, making them highly reliable and long-lasting devices ^2^. High energy efficiency technology of Fuel cells is mainly powered by H2 generated from secure and renewable sources, while operation by-products are water and CO _2_ emissions. Hydrogen possesses an energy capacity of 122 kJ/g (2.75 times higher compared to numerous hydrocarbon fuels) and can store 2.6 times more energy than gasoline ^3^. Therefore, H _2_ is an ideal energy carrier and gets growing interest to satisfy the energy needs. FCH technologies could play a significant role to meet the energy requirements of the modern world in an environmentally friendly and permanent way. Moreover, unlike conventional energy accumulation/transformation devices (such as batteries and internal combustion engines), fuel cell technologies eliminate the costs related with handling/storing toxic materials such as battery acid or diesel. Acidic polymer electrolyte membrane (PEM) and alkaline (AEM) fuel cells are the most promising technologies and have already been used in automotive market (TOYOTA Mirai PEM fuel cell vehicle) and space travels (Gemini and Apollo missions). Today, more than 30,000 passenger fuel cell vehicles are on the world’s road, while the STELLANTIS Group had announced plans to return to fuel cell vehicle production and commercialization by the end of 2021. Nevertheless, a significant volume of PEM fuel cells will reach their end-of-life by 2030, and currently there are not cost efficient processes for recycling their valuable parts containing PGMs. Apart from H _2_ fuel storage issues, another serious drawback that fuel cell cars have not yet reached a massive production and selling level, is their high cost ^4^. It should be mentioned that a TOYOTA Mirai has x8 times higher cost than the corresponding passenger car with internal combustion engine. This is partially attributed to the Platinum Group Metals (PGMs) content and price, since a fuel cell car contains 30–60 g of PGMs, while the automotive catalyst contains a total of 3–5 g of PGMs (depending upon the type of the vehicle). The EU area consumes the world’s largest volume of PGMs in industrial applications; ca. 20% of demand worldwide ^5^, with ~ 90% of the Pt and 54% of the Pd supply channelled to the production of (electro)catalysts, making it the most demanding application ^6^. Therefore, the main challenge with the current situation is twofold: i) PGMs prices are rising rapidly and the specific materials are facing a serious supply shortage as the PGMs market has been in deficit since 2019, and all-time highs were seen in PGMs prices in 2020, and ii) the almost not existent production of PGMs in Europe ~84% of the primary supply is imported from South Africa and Russia, which have suboptimal geopolitical stability, making Europe entirely dependent on this import ^5^. Thus, PGMs are continuously on the Critical Raw Materials List of the EU since 2011 (updated in 2014, 2017 and 2020), with increasing criticality towards EU exploitation ^7^.

Based on the above, it is imperative for the EU to secure the supply of PGMs for its end-use applications, allowing room for prospective organisations to be created and operate profitable business on the specific activity. On this scope, the recycling of PGMs containing EoL materials and their reutilisation in the production of new ones, without compromising of the performance and operating efficiency of the new devices, is the major route for reducing the EU PGMs supply/demand gap. In this paper, the technoeconomic aspects of recycling and re-manufacturing of electrocatalysts are analyzed. This is done by applying fundamental business analysis elements adapted to innovation concepts, to overly indicate the prospective business operation margins under the current supply of EoL sources. 

## Methods

The innovation Go-to-Market strategy includes traditional marketing tools and financial sustainability indicators combined and redefined for commercializing offerings (processes and products). In this framework, the elements below are utilised for the aforementioned study:

• ***Market and Competition Analysis***, focusing on the current status and size of the market, market trends and expansion potential, the key/dominant players, existing offerings and research under development, categorised depending upon the target TRL of the innovative offering.

• ***Company Go-to-Market strategy development***, initially formed by utilising a SWOT analysis to identify the Strengths and Opportunities of the market with regard to the S/T development and commercialisation of the offering. Further, possible Weaknesses and Threat in this aspect are identified and mitigation measures are formed to minimize risks and accelerate the commercialisation process.

• ***Business Model Canvas*** to define the business model for generating maximum value to the involved stakeholders as determined by the value proposition of the innovative offering, minimizing in parallel production and commercialisation costs. Special attention is given in the creation of Circular Business Models taking into consideration environmental and social aspects during the commercialisation and ensure an effective business model with minimal socioenvironmental impacts.

• ***Market Share projections***, including a bottom-up financial modelling forecast considered for the short term, usually the beginning of the commercialisation and up to 5 years ahead, while the commercialisation process is stabilising, and the resource (materials sourcing) capacity is established. Beyond this point, a top-down method is used to determine the financial projections based on market share and commercialization potential (Total Available Market-TAM, Serviceable Available Market-SAM, Serviceable Obtainable Market-SOM model).

• ***Financial planning***, depicting all the commercialization stages as the business potential is evolving, together with revenues and costs streams related with the offering commercialisation in a cumulative Profits and Losses (P&L) table. With respect to innovative market offerings, fundraising rounds are also included and ultimately the financial sustainability indicators are calculated: Net Present Value (NPV), Initial Rate of Return (IRR) and the Break-Even Point for the investment made.

## Results

### Market analysis

***PGMs market:*** The global precious metals market, in which PGMs are included together with Au and Ag, was valued at EUR152B (2019) in revenue and the Compound Annual Growth Rate (CAGR) being at 9% between 2020–2027. Besides fuel cells (utilizing Pt) and electrolysers (utilising Ir, Ru, Pt, Pd), PGMs are used in a wide range of commercial applications (e.g. electronics, automotive catalytic converters, medical applications, etc.) for which there are often no substitutes. In 2019, the global PGMs supply was 447 tn, out of which 180 tn for Pt and 210 tn for Pd, while the rest accounted for 57 tn. The corresponding gross demand for applications reached 679 tn, creating an annual supply-demand gap of 232 tn. The EU area consumes the world’s largest volume of PGMs in industrial applications; ca. 20% of demand worldwide Europe is currently the world’s largest consumer of PGMs in industrial applications with a share of ca. 20% of the global demand, estimated at ~154 t worth ~7.5B with current PGMs prices. There are no primary production sites of PGMs in the EU area, and approximately 84% of the primary supply comes from South Africa and Russia. Only about 15% of the worldwide PGMs supply is sourced by processing secondary resources (catalysts, jewellery, high-grade PCBs), while mining of primary deposits, presents extensive operational costs and environmental footprint. In the European area, the market is dominated by 7 key companies (Johnson Matthey-UK, KaiDa Technology-UK, Umicore-BE, Chimet S.p.A.-IT, BASF-DE, Evonik-DE, Heraeus- DE), with Johnson Matthey having ca 40% of the EU market. However, there is a current supply/demand deficit and Brexit hardens the commercial networks of JM in the EU. The majority of the large recycling companies (e.g. Umicore, Johnson Matthey, BASF) apply pyrometallurgical processes for the treatment of PGMs sources and their recovery, which suffer from high energy consumption, high environmental footprint and adverse impacts to both human health and environment, such as those caused by Pb-collectors in the smelting process.

***Fuel cells market:*** The fuel cells market is valued at EUR 218M (2020) and is projected to reach EUR 703 by 2025 (GAGR 26%). The highest end-use case is transportation with more than 30,000 fuel cell cars on the roads, globally. With zero emissions perspective, this market is expected to reach 600,000 units until 2032. Taking into account that each commercial fuel cell utilized in electric car contains about 30–60 g Pt, high demand will be accumulated for Pt for this application in the near future. Automotive proton-exchange membrane fuel cells (PEMFCs) have reached sufficient development to be commercially utilised by major automotive companies (incl. TOYOTA, Honda and Hyundai) in electric vehicles. Hydrogen technology cars offer many advantages over battery electric vehicles such as: refuelling time at ca. 3–5 min, large milage range-up to 600 km between refuelling, extended lifetime (>200,000 km), high-comfort and safety while driving. Nevertheless, the main residual challenge for PEMFCs is cost. The increased cost of hydrogen cars is due to high mass of platinum (Pt) catalysts being used and the limited production volumes of these devices. Even though Pt loading has decreased significantly in the last few years to ca 0.15 mg Pt/cm ^2^ in MEAs, it is a major area needing further reductions. Daimler has reached a 90% reduction of Pt loading in hydrogen models (Mercedes GLC F-Cell vs B-Class F-Cell) since 2009, while Toyota is aiming for a 50% reduction in the TOYOTA Mirai.

Furthermore, fuel cells have been proven as a cost effective solution for secondary power generation, especially for the telecommunication field. Batteries and generators that operate on carbonaceous fuels are utilised in most backup-power communication and control systems to allow for undisrupted connection. These traditional power systems are widely utilised, while the need for cost efficient reliability and durability are the main drivers for the industry to seek other power generation technologies. In comparison, fuel cells can offer extended lifetime and better performance in non-optimum environmental conditions. Another advantage of this technology is the compatibility, where fewer moving parts are incorporated, thus requiring less maintenance. Besides, fuel cells systems allow for significant operational cost reduction as they have shorter runtime cycles (max 72 hours operation), compared to conventional battery battery/batterygenerator systems. However, the purchasing cost of a fuel cell power generator is higher upfront, as mentioned before, mainly due to high Pt utilization in MEAs. Under the conventional manufacturing methods, it is projected that ultralow mass utilisation of Pt coupled with large production capacities of fuel cells, could drastically reduce the associated manufacturing costs over the next 10 years. 

***Electrolysers market:*** The electrolysers market is currently valued at EUR 153M (2020) and is projected to reach EUR 257M by 2026 (GAGR 9%). Electrolysers are primarily utilized in electricity production units and is applicable in the automotive industry, in pharmaceutical and biotechnology and metals and glass industries. Marketwise, the specific market is expected to increase by at least 1000 times by 2040 (from 200 MW current capacity), with 200 GW of electrolyser facility projects in the making, driven by a highly demanding European industrial ecosystem, with infrastructure of over 20 GW installed in Germany, the Netherlands, and Britain, by the end of 2030. With the projected capacity of 214 GW, the EU area could generate up to 32M tn of hydrogen per year, meeting at least 50% of the current demand. Besides, electrolyser facilities are rapidly scaling up, with plans to upgrade the 1–10 MW facilities to nominal power capacities of 100–500MWs by 2024, to supply local ecosystems. The EU aims to have 40 GW of electrolyser projects operational by 2030, while governments have already initiated projects totalling 34 GW by that date. However, it has been recognized by experts that such an ambitious target cannot be implemented without significant costs reduction in electrolyser infrastructure. PEM electrolysers utilize PGMs (mainly Pd, Ir, Ru, Pt) for their operation, with their EU supply being at limited levels compared to the projected demand. 

### SWOT analysis

For the purposes of understanding and evaluating the status of the specific market and its potential in relation to recycling and electrocatalysts re-manufacturing, the SWOT analysis was formed ( Table 1).

**Table 1.** SWOT analysis.

**Strengths**	**Weaknesses**
S1) Knowledge and expertise on the (raw) materials field and recycling technologies established by research activities for fuel cells and electrolysers, particularly using hydrometallurgical means. S2) Novel, low cost, high efficiency processes, tested for the particular materials (MEAs), exist already in the industrial sector. S3) PGMs recycling is aligned with environmental strategies. S4) PGMs are critical raw materials for the EU and have finite sources worldwide. S5) Robust circular economy operation is created and sustainability is promoted.	W1) As the fuel cells/electrolysers market has recently been established, limited EoL materials exist, thus production capacity will not be sufficient to gain significant market share. W2) Commercial channels are not widely established for recycled materials, especially when high specification products are needed. W3) Business expansion and industrialization needs extensive funding.
**Opportunities**	**Threats**
O1) Green energy production markets are expected to skyrocket in the next few years and the respective devices will be widely utilised across different markets. O2) Public/Private opportunities for funding for further development and business expansion exist in the EU and worldwide. O3) Environmental regulations demand a transition to green energy production across major fields worldwide. O4) The recycled materials are competitive in terms of technical characteristics and price.	T1) The PGMs market is highly competitive-consolidated market and difficult to penetrate T2) Market mistrust to recycled and novel products by customers and end users. T3) Product customization and high purity is needed for certain applications, e.g. electrocatalysts for fuel cells/electrolysers and catalysts for automotive catalytic converters.

The strengths and opportunities identified allow solid recycling and electrocatalysts manufacturing business to be operated for green energy production devices. The advantages of hydrometallurgical processes over pyrometallurgical indicate the preferred use of the specific technology for this business operation. On the other hand, weaknesses and risks have been identified to help resolve marketability issues and deal with bottlenecks to stay ahead of the market. Specifically, mitigation plans are developed for T2 and T3 as process/products demonstrations shall be conducted and relevant certifications need to be obtained for the products and their specifications to match market needs, according to end-products manufacturers. Coupled with prospective dedicated marketing activities in the specific sector, rapid and wide awareness will be created to inform the targeted stakeholders of the value proposition(s) and the quality of the recycled offerings over conventional ones, as well as the potential business opportunity (T1). The mitigation plans for the weaknesses are discussed in the financial planning section.

### Business model canvas

**Figure 1.**
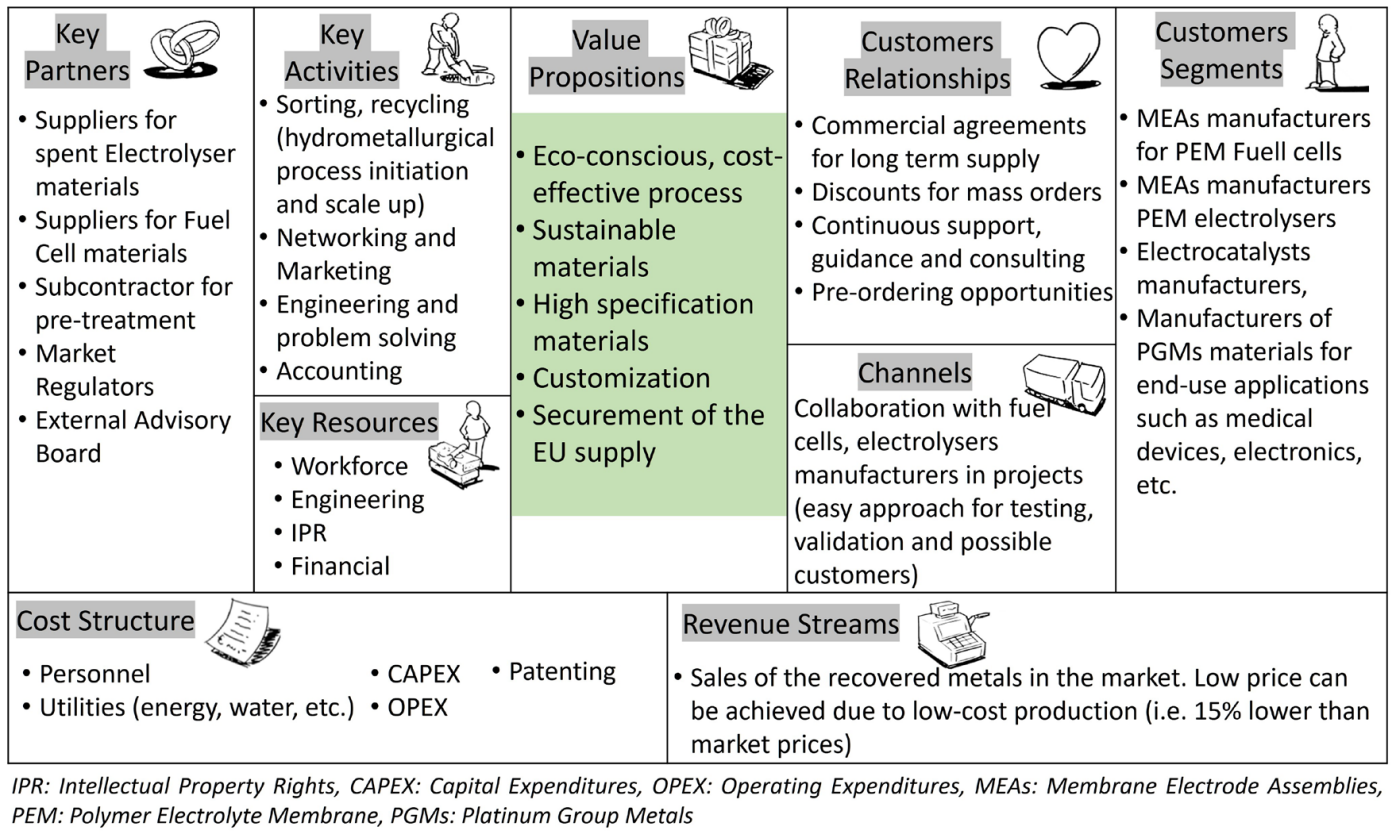
Business Model Canvas.

Based on the market analysis and the SWOT analysis, the Business Model Canvas is structured to identify the key elements regarding the business model to be followed towards products commercialisation ( Figure 1).

The business model that was deemed optimal for operating a materials recycling/electrocatalysts re-manufacturing business under the current condition of the market is defined in the Business Model Canvas ( Figure 1), centred by the Value Propositions of the novel hydrometallurgical process and the recycled products. Suppliers of EoL green energy production devices (electrolysers and fuel cells) will deliver materials, either the whole device or series of devices namely stacks, where pre-treatment can be subcontracted for business inclusiveness and commercial channels expansion, or parts of the device, such as the MEAs, to the recycling facilities to be treated in premises of the company. There, the innovative hydrometallurgical process for PGMs recovery will take place where finally, the recovered PGMs powder will form the intermediate products (Pure Metals and Metal alloys, as well as Nano Metal Oxides) that will be used to form the final new electrocatalysts materials.

The revenue stream is based on selling electrocatalysts for MEAs manufacturing for PEM automotive fuel cells and electrolysers for their utilisation in MEAs and PEMs fuel cells for light duty passenger cars (e-mobility) and green energy power stations. Their estimated price will be formulated according to market prices, the loading of the precious elements, and also taking into account manufacturing costs. Since the raw materials used will derive from a recycling process, their prices will exhibit a ca. 15% cost reduction compared to commercial ones. For reference, it is estimated that the sale price of the recycled electrocatalysts will range at EUR 14.5/g Pt/C, EUR 17/g Pd/C, EUR 21.3/g RuO2, EUR 128/g IrO _2_. This in turn will allow for a cost reduction in the production of MEAs and automotive fuel cells (MEAs coated with electrocatalysts represent ca 60% of the production cost of one fuel cell device). Therefore, fuel cells with recycled MEAs will also present a ca 10% cost reduction, making them more affordable for the automotive sector, to be utilised in the future vehicles fleet.

### Financial plan

Based on the aforementioned business model and the status of the market, a combined 5-year financial plan was drafted for the PGMs recycling revenue stream, to illustrate the business and profit margins coming from the respective sales ( Table 2 ^8^) as evaluated from the current status of the market for the period 2019–2021. The financial study and planning were conducted from a conservative to moderate point of view to indicate the low end of commercialisation financials.

**Table 2.** Profits and losses (P&L).

*(All values in EUR)*	Year 1	Year 2	Year 3	Year 4	Year 5	Total
**EoL MEAs recycled (piece)**	**331200**	**592000**	**920000**	**1576000**	**3120000**	**6539200**
**Electrocatalysts quantity for sale (kg)**	**28**	**48**	**74**	**126**	**221**	**498**
Pt/C	19	36	56	97	162	**370**
Pd/C	7	8	12	21	42	**90**
RuO2	2	2	3	5	10	**21**
IrO2	1	2	2	4	8	**17**
**Revenues -**15% off the market prices (14450 €/kg Pt/C, 17000 €/kg Pd/C, 21290 €/kg RuO2, 128350 €/kg IrO2)	**579724**	**912505**	**1397657**	**2367962**	**4273523**	**9531370**
**Other Revenue streams (private/public fundrainsing)**	**60000**	**60000**	**50000**	**50000**	**50000**	**270000**
**Total Gross Revenue from sales**	**639724**	**972505**	**1447657**	**2417962**	**4323523**	**9801370**
**CAPEX**	**1500000**	**0**	**0**	**0**	**0**	**1500000**
Dissolution, filtration units	500000	0	0	0	0	500000
Solvent extravtion unit	400000	0	0	0	0	400000
Recovery unit	300000	0	0	0	0	300000
Electrocatalytsts manufacturing unit	300000	0	0	0	0	300000
**OPEX**	**567806**	**614029**	**679209**	**784588**	**1040644**	**3686276**
Materials (inc EoL MEAs @0.15 EUR/cm ^2^, C, consumables, etc.)	89680	128800	178000	276400	508000	1180880
Staff Cost (3 employees full time)	242640	242640	242640	242640	242640	1213200
Operations (incl energy, engineering, installation, etc)	65486	72589	88569	95548	120004	442196
Decreciations (10% of CAPEX)	150000	150000	150000	150000	150000	750000
Marketing	20000	20000	20000	20000	20000	100000
**Total Cost**	**2067806**	**614029**	**679209**	**784588**	**1040644**	**3686276**
**Other Indirect Costs (10% of Total Costs)**	**206781**	**61403**	**67921**	**78459**	**104064**	**6191672**
**Operating Profit**	**-1634863**	**297073**	**700527**	**1554915**	**3178815**	**6115094**
**Tax (30%)**	0	89122	210158	466474	953644	1719399
***Total Net Profit***	***-1634863***	***207951***	***490369***	***1088440***	***2225170***	***4395695***

It is noted that for evaluating the CAPEX and OPEX of the business case with regard to the maximum EoL materials collected and treated by the process, a hypothesis of a new hydrometallurgical plant is assumed in the scale of semi-industrial, sufficient to treat the below mentioned capacities. The associated infrastructure considered consists of the basic elements mentioned below, representative to a real industrial facility for treating the generating EoL sources: a) three leaching reactors of min 1000 litres volume (estimated price EUR 300,000)

b) a filtration unit with a minimum of four filtering columns (estimated price EUR 200,000)

c) a mixer settler battery for separation of streams (estimated price EUR 400,000)

d) a precipitation/electrorecovery tank (estimated price EUR 300,000)

e) a large scale automated electrocatalysts sputtering machine (estimated price EUR 300,000)

The associated operating costs for running the unit are estimated to range from EUR 0.5 to 1 million depending upon the expansion of the unit and the processing capacities. It is noted that, at this stage a semi-industrial facility was considered due to the limited resource availability of the EoL materials, and an evaluation was made for minimizing CAPEX/OPEX costs, while maximizing the treatment capacity of the related infrastructure. Overall, the initial upfront investment is estimated at EUR 2 million, while an emergency reserve is estimated at EUR 0.5 million. A semi-industrial unit can be operated with ca 3 full time employees costing an average of EUR 240 per day (based on an 8-hour shift), estimated based on the high end of an average EU salary for specialized personnel.

The business plan will be implemented in the beginning of the commercialisation, with only limited number of products that will be produced. The upscaled production line will be ready to recycle ca. 300k ΕοL MEAs annually from year 1 to produce ca 30 kg of electrocatalysts, gradually reaching 3 million MEAs recycled and 200 kg PGMs produced, in year 5. Market shares will gradually increase with primary target the EU PGMs market (Serviceable Available Market-SAM) and at second level the global market (Total Available Market-TAM). By year 5, the business dynamics reflect on obtaining ca 0.5% of the EU market (Serviceable Obtainable Market-SOM) (W3). This will have a significant impact on prospective revenues and profit, which can be utilised towards further investments in case of a good commercialisation scenario to further upscale the production (W2). Furthermore, it is estimated that due to the innovative nature of the business, funds from private/public investment opportunities (e.g. Venture Capitals, EU Horizon/EIT KIC projects, Angel Investors) can be secured, as well as reinvestments of the revenue reserves for business expansion once more materials can be available for recycling. Based on conservative/moderate projected recycling capacities depicted in Figure 1, the foreseen revenues translate into a Net Present Value (NPV) of EUR 1.7 million (WACC 5%) with an Initial Rate of Return of 32%, and payback period of 3.8 years, making the specific commercialisation scenario highly attractive for investors, with high implementation potential.

## Conclusions

The green energy production sector is currently under expansion under recent regulations and plans from the EU (e.g. Paris Agreement, Green Deal) for carbon neutrality until 2050. This allows for the development of novel technological devices fuel cells and electrolysers to have a catalytic role in green energy production in the near future. However, such devices utilize PGMs for their operation which have been characterized as critical by the EC. The business opportunities arising from this challenge refer greatly in the recycling of EoL fuel cells and electrolysers to reutilize the PGMs contained in them into new devices. Aiming for a profitable commercialization scenario, the industrials sector must operate a unique processing technology, coupled with Innovation Business Planning to success in commercializing the products. As a simplistic business case, a novel hydrometallurgical process was theorized, potentially sprouting from existing Research and Innovation (R&I) activities as is usually happening with EU cross collaborative projects. The associated marketability elements (Business Model Canvas, SWOT analysis and Financial Planning) can solve major bottlenecks in the beginning and the expansion of a prospective business, and in particular in case of start-up companies. A case study scenario for the recycling of PGMs from EoL fuel cells and electrolysers in the EU area showed that there is potential for profitable operation, even under the conservative/moderate assumptions made for the production capacities and the interest of the markets. Further, it was shown that in such case, the PGMs production business can be decentralized from the few key players dominating it and revolutionized by novel processing methods (i.e. hydrometallurgical over conventional pyrometallurgical ones). On this scope, the respective upfront investment was estimated at EUR 2.5 Million to include all major infrastructure elements alongside the operating costs for installing and operating the unit, while novel processing routes through hydrometallurgy can allow for price reduction of the final products of ca 15%. This in turn, will reduce the cost of commercial electrolysers and fuel cells, making them more affordable for wide utilization and everyday end-use applications. In turn, low cost fuel cells will contribute to lowering the price of fuel cell cars (ca EUR 50,000 current average price) to the EUR 30,000 price range and making them cost competitive with regard to commercial petrol/diesel ones. As concluded from the business case, the initial investment for a semi-industrial recycling/manufacturing facility (ideal balance between cost efficiency and processing capacity maximization) can be repaid within a maximum of 4 years and be financially sustainable in the long term with positive NPV and IRR. Finally, taking into account, societal and environmental aspects, such business scenario very well complies to reduce technological residues and improve societal aspects such as jobs creation, infrastructure and raw materials expertise reinforcement.

## Data availability

Open Science Framework: PROMET_H2, https://doi.org/10.17605/OSF.IO/UZ4NH ^8^.

Data are available under the terms of the Creative Commons Zero "No rights reserved" data waiver (CC0 1.0 Public domain dedication).

Revenue projections were made based on the market analysis on the global vehicles fleet powered by fuel cells (30,000 fuel cell cars currently on the roads and expected to reach 600,000 units until 2032). It was assumed that 1,6% of the respective fleet reaches EoL per year, based on their estimated lifespan (100,000 km warranty). Taking into account that each fuel cell contains ca 400 MEAs and 30 g of Pt in total, the associated revenues were calculated based on recycling the PGMs and selling them at discounted prices (15% less) compared to market, as noted in Table 2. The same methodology was followed for calculating the MEAs volume for recycling coming from EoL electrolysers. The projections were made according to market data for 320 electrolyser units in the EU area, taking into account that the recovered PGMs are sold at 15% less than market prices and based on the recovered volumes. The price reduction arises from the novel concept of recycling EoL sources and the associated reduced CAPEX/OPEX hydrometallurgical process operate under. Finally, considering the latter, fundraising rounds can be made possible to exploit the benefits of such concept at the fullest. 
