# Supplementary material for: Recycling of platinum group metals from energy storage devices: a techno-economical business plan analysis
Source: Open Res Eur. 2022 Jul 27;2:92. [Version 1] doi: 10.12688/openreseurope.14866.1 (PMC10446025; doi:10.12688/openreseurope.14866.1)
Supplement: Peer review history

# Peer review: Reviewer response for version 1

**DOI:** 10.21956/openreseurope.16061.r29789

**Published:** 2022-08-15

**Authors:** Sergio Rochas

**Affiliations:** 1 Sustainable Energy and Chemistry (EQS), Instituto de Catálisis y Petroleoquímica, Madrid, Spain

The manuscript by S. Spathariotis *et al.* addresses a fundamental aspect, although rarely covered, of the green economy, namely the imperative need of recycling PGM (Pt, Ir, Pd, Ru…) from the devices used for the production and use of green hydrogen. In particular the authors focus on the recovery of Pt from fuel cells, especially the fuel cells used in the automotive sector, and the Pt, Ir and Ru used in electrolyzers. The importance of this feature cannot be overestimated, since mining of PGM alone will not suffice to satisfy EU’s expected demand of these metals. In addition, as properly identified by the authors, the largest reserves of PGMs, and some of the main companies controlling PMG markets are located in third countries.

 In their work, the authors present a technoeconomic analysis of the recovery and recycling of PGM metals making a case study for the hydrometallurgical, which according to the authors is superior to the pyrometallurgical one. A brief description of each process should be necessary in order to understand the key aspects of each process, emphasizing the advantages of the hydrometallurgical process. Do the authors know if this process is currently used at commercial level?

 Through the manuscript, some figures are given without citing references to support them. For instance, it is said that the sale price of the recovered materials could be 15% lower than current market prices. How do the authors get this figure? It is stated that between 30-60 g of Pt are used in fuel cell vehicles. This figure may be a little lower, between 10-20 g (see Thompson *et al.* (2019 ^1^), or SAE Int. J. Sust. Trans., Energy, Env., & Policy / Volume 3, Issue 1, 2022).

 In their case study, the authors aim to recover Pt, Pd, Ru and Ir from MEAs. What kind of MEAs use Pd?

 It appears that three persons will be hired to process ca. 6.5 million MEAs in 5 years. This is more than 1200 MEAs per day. Is this figure correct?

 In the list of equipment an automatic electrocatalyst sputtering is found? Is this equipment needed for preparing the electrocatalysts or for preparing MEAs?

If applicable, is the statistical analysis and its interpretation appropriate?

I cannot comment. A qualified statistician is required.

Is the study design appropriate and is the work technically sound?

Yes

Is the work clearly and accurately presented and does it engage with the current literature?

Yes

Are the conclusions drawn adequately supported by the results?

Yes

Are sufficient details of methods and analysis provided to allow replication by others?

Partly

Are all the source data and materials underlying the results available?

Yes

Reviewer Expertise:

Synthesis, characterization and testing of electrocatalysts for fuel cells and electrolyzers

I confirm that I have read this submission and believe that I have an appropriate level of expertise to confirm that it is of an acceptable scientific standard.

## References

[1] : Platinum group metal-free catalysts boost cost competitiveness of fuel cell vehicles. *Nature Catalysis* .2019;2(7) : 10.1038/s41929-019-0291-x 558-561 10.1038/s41929-019-0291-x

# Peer review: Reviewer response for version 1

**DOI:** 10.21956/openreseurope.16061.r29777

**Published:** 2022-08-08

**Authors:** Andreas Benardos

**Affiliations:** 1 School of Mining and Metallurgical Engineering, National Technical University of Athens, Athens, Greece

The work presented by the authors is of good quality and deals with a very interesting research topic, regarding the recycling potential of PGMs from energy storage devices.

 The importance of recycling platinum group metals is stressed in this work and the economics are calculated for a prospective operating business. The authors have included a very well structured market analysis focusing on the main PGMs containing devices and have presented thoroughly the current status of the corresponding markets, the needs and the main drivers for wide utilization and exploitation.

 The methodological analysis the authors implemented for extracting meaningful results is well described and consists of basic elements of a business plan analysis. The adaptation of the business plan analysis elements is relatively thorough and financial data and analysis is presented to support the assessment made.

 Overall, the presented work is of good quality and addresses an emerging application with high potential for market deployment. The data extracted can be useful from a business operational perspective for the industrial ecosystem. However, there some additional points that require further clarification in an attempt to further support the decisions and assumptions made as well as to further enhance the quality of the manuscript.

 To this end the following are stated:

1. Important details regarding the market analysis which is presented in the manuscript are given without having being cited appropriately. The authors are encouraged to address this particular issue with proper referencing of the information obtained and used, while they also should stress and identify their own primary market research, if any.
2. In the SWOT analysis made in Table 1 the authors accurately state the alignment of the proposed effort with the EU strategies already in place. However, issues like minimizing resource dependence in the PGMs and promoting resilient raw material supply chains are not that clearly stated (contrary to the given business model canvas - BMC). Thus, this particular issue should be further commented on and analysed in the manuscript.
3. The authors are referring to the advantages of hydrometallurgical processes over pyrometallurgical ones. Also they state that this is the preferred technology for their approach. Nevertheless, this is not covered with additional details and although the financial plan section gives brief info regarding the materials/equipment used, the authors should clearly define the hydrometallurgical processes and concept used before the initiation and presentation of the BMC.
4. The authors' attention is required in Table 2 to clarify the values given (and commented within the manuscript body):
  (a) First of all it is advised that it should be re-written using digit grouping format (perhaps with the use of values in 1,000 € format), allowing the reader to clearly understand the data and numbers given.
  (b) The final column giving the aggregation of the categories should be changed as CAPEX and OPEX data are aggregated which is not standard practice in the DCF analysis. If the values are presented, after their discounting (or with the use of WACC), this can be made but only in the OPEX data. After all the overall combined and discounted value of the project is given through the NPV value.
  (c) Are the Depreciation and Amortization taken into account? There is a "Decreciations" category that appears to express general expenses but not any (linear?) depreciation of the CAPEX.
  (d) Does the "total net profit" column express the yearly cash flow of the project?

If applicable, is the statistical analysis and its interpretation appropriate?

Yes

Is the study design appropriate and is the work technically sound?

Yes

Is the work clearly and accurately presented and does it engage with the current literature?

Yes

Are the conclusions drawn adequately supported by the results?

Yes

Are sufficient details of methods and analysis provided to allow replication by others?

Yes

Are all the source data and materials underlying the results available?

Yes

Reviewer Expertise:

Mining and Metallurgical Engineering; Cost estimation and feasibility assessment of projects; Design of Underground Mining and Construction projects; Risk Analysis;

I confirm that I have read this submission and believe that I have an appropriate level of expertise to confirm that it is of an acceptable scientific standard.
